# Preoperative lymphocyte‐to‐monocyte ratio is useful for stratifying the prognosis of hepatocellular carcinoma patients with a low Cancer of the Liver Italian Program score undergoing curative resection

**DOI:** 10.1002/ags3.12251

**Published:** 2019-04-23

**Authors:** Takayuki Shimizu, Mitsuru Ishizuka, Kyung Hwa Park, Takayuki Shiraki, Yuhki Sakuraoka, Shozo Mori, Yukihiro Iso, Masato Kato, Taku Aoki, Keiichi Kubota

**Affiliations:** ^1^ Second Department of Surgery Dokkyo Medical University Tochigi Japan

**Keywords:** CLIP score, curative resection, hepatocellular carcinoma, low CLIP score, lymphocyte‐to‐monocyte ratio

## Abstract

**Background and Aim:**

Although the Cancer of the Liver Italian Program (CLIP) score is useful for prognostication of patients with hepatocellular carcinoma (HCC), a previous study has reported that the CLIP score was unable to stratify the postoperative outcomes of HCC patients in whom the score was low (0‐1). Recent studies have reported that the preoperative lymphocyte‐to‐monocyte ratio (LMR) is useful for prognostication of patients with various cancer.

**Methods:**

We reviewed 329 HCC patients with a low CLIP score (0‐1) undergoing curative resection. This study had the approval of the Institutional Review Board (28068). Multivariate analyses were carried out to detect clinical factors correlating with overall survival (OS). Kaplan‐Meier analysis and the log‐rank test were used for comparison of OS.

**Results:**

Multivariate analysis showed that LMR (<4.35/≥4.35) was significantly associated with OS (hazard ratio [HR], 2.022; 95% CI, 1.141‐3.583; *P *=* *0.016) as well as portal vein invasion (HR, 2.410; 95%CI, 1.258‐4.618; *P *=* *0.008). Kaplan‐Meier analysis and the log‐rank test showed a significant difference in OS and relapse‐free survival between patients with high LMR and those with low LMR.

**Conclusion:**

Preoperative LMR is useful for stratifying the prognosis of HCC patients with a low CLIP score (0‐1) undergoing curative resection.

## 1 INTRODUCTION

Prognosis of hepatocellular carcinoma (HCC) patients may not be fully predictable on the basis of tumor‐node‐metastasis (TNM) stage alone. However, the Child‐Pugh classification, which represents liver functional reserve, is able to stratify the prognosis of such patients.^1^ In order to provide better prognosis of HCC patients, the Cancer of the Liver Italian Program (CLIP) score, the Japan Integrated Staging (JIS) score and the Tokyo score were established as an integrated prognostic system for HCC patients, combining tumor progression with liver functional reserve.^2^, ^3^, ^4^ Previous studies have reported that the JIS score seems to be a better predictor for prognosis of patients undergoing liver resection in comparison with the CLIP score and the Tokyo score.^5^, ^6^ Recently, a large population study (n = 3182) showed that the CLIP score is the best prognostic system for HCC in comparison with several prognostic systems including the JIS score and the Tokyo score, because the CLIP score had the highest homogeneity in patients with the same stage.^7^ This study showed that the CLIP score attracted attention as the best predictor of HCC patients. However, the CLIP score inadequately estimates outcome after curative resection for HCC, because it includes HCC patients with distant metastasis (3.2%, 14/435), Child‐Pugh classification C (15.9%, 69/435), and non‐surgical treatment (90.8%, 395/435).^2^

Although a previous study has reported that the CLIP score is useful for prognostication of HCC patients receiving non‐surgical treatment,^8^ it is inferior to other prognostic systems for prognostication of HCC patients undergoing surgery.^5^ In our previous study, most HCC patients (74.0%, 222/300) undergoing liver resection had a low CLIP score (0‐1).^9^ Additionally, because CLIP score was not good at stratifying the postoperative outcome of such patients, CLIP score was inferior to hepatic Glasgow Prognostic Score in prognostication of HCC patients undergoing liver resection.^9^ In order to apply the CLIP score to prognostication of HCC patients undergoing curative resection, further investigation is required to stratify the prognosis of those with a low CLIP score.

Recent studies have shown that the lymphocyte‐to‐monocyte ratio (LMR) is useful for prognostication of patients with various types of cancer.^10^, ^11^ LMR includes lymphocytes and monocytes, which play a crucial role in the immune system. As tumor infiltrating lymphocytes are well known to exert an antitumor effect by inhibiting the proliferation of tumor cell, decreased lymphocyte count might indicate a weak antitumor reaction and poor clinical outcome.^12^ In contrast, monocytes are white blood cells that can further differentiate into a range of tissue macrophages and dendritic cells.^13^ Monocytes promote tumorigenesis through local immune suppression^14^ and, in addition, can differentiate into tumor‐associated macrophages (TAM), which promote tumor growth, angiogenesis, invasion, and metastasis.^15^ Thus, in cancer patients, peripheral blood lymphopenia and an increased monocyte count are associated with poor prognosis.

In patients with HCC, several studies have reported that the LMR is significantly associated with surgical outcome after curative resection.^16^, ^17^, ^18^ As a low LMR was associated with tumor size, vascular invasion and tumor staging, it could be a good predictor of post‐surgical outcome in HCC patients.^16^, ^17^, ^18^ Therefore, LMR might be able to stratify the prognosis of HCC patients with a low CLIP score undergoing curative resection. In the present study, we investigated whether the LMR was able to stratify the prognosis of HCC patients with a low CLIP score.

## 2 PATIENTS AND METHODS

Three hundred and twenty‐nine patients who underwent initial and curative resection between April 2005 and December 2015 at our department were retrospectively reviewed. All operative procedures had been carried out by the same surgical team at Second Department of Surgery, Dokkyo Medical University Hospital. All patients in the present study had a preoperative low CLIP score (0‐1). Patients who underwent non‐curative surgery, surgery for HCC recurrence, or combined resection of other organs were excluded. There were no patients who had infectious disease, chemotherapy or irradiation therapy before surgery for HCC.

**Figure 1:**
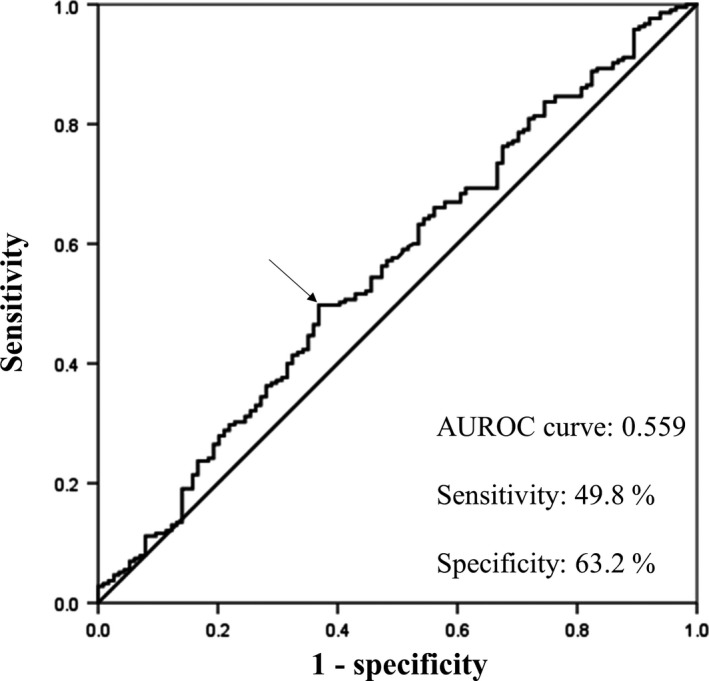
Receiver operating characteristic (ROC) curve shows the optimal cut‐off value for the lymphocyte‐to‐monocyte ratio (LMR). Arrow shows the most prominent point on the ROC curve. AUROC curve of the LMR for overall survival is 0.559

Using the Youden index (maximum value of [sensitivity – (1 − specificity)]),^19^ we determined the cut‐off value of variables such as alpha‐fetoprotein (AFP) (ng/mL), age (years), alanine aminotransferase (ALT) (IU/L), aspartate aminotransferase (AST) (IU/L), indocyanine green retention rate at 15 minutes (ICGR15) (%), maximum tumor size (cm), platelet count (×10^4^/mm^3^), protein induced by vitamin K antagonist II (PIVKA‐II) (mAU/mL), prothrombin time (PT) (%), total bilirubin (mg/dL) and white blood cell (WBC) count (×10^3^/mm^3^), except for serum levels of albumin (g/dL) and C‐reactive protein (CRP) (mg/dL). Upper limits of normal serum levels of albumin (3.5 g/dL) and CRP (0.3 mg/dL) at our institution were defined as the cut‐off values for albumin and CRP, respectively. The recommended cut‐off value for the LMR was 4.35. LMR predicting for overall survival (OS) had a sensitivity of 54.8%, specificity of 64.6%, and an area under the ROC (AUROC) curve of 0.559 (Figure 1).

### 2.1 Calculation of LMR

Lymphocyte‐to‐monocyte ratio was calculated as: LMR = peripheral lymphocyte count (/mm^3^ or %)/peripheral monocyte count (/mm^3^ or %).

### 2.2 Diagnosis of liver cirrhosis

Liver cirrhosis was pathologically diagnosed as stage f4 when bridging fibrosis was observed surrounding the regenerative nodules in the liver parenchyma of the resected specimen.^20^

### 2.3 Pathology of HCC

Pathological differentiation was diagnosed on the basis of the General Rules for the Clinical and Pathology Study of Primary Liver Cancer (Liver Cancer Study Group of Japan, 3rd English edition).^21^

### 2.4 Diagnosis of portal vein invasion and hepatic vein invasion

Portal vein invasion and hepatic vein invasion were diagnosed on the basis of the General Rules for the Clinical and Pathological Study of Primary Liver Cancer (Liver Cancer Study Group of Japan, 3rd English edition).^20^

### 2.5 Definition of CLIP score

The CLIP score is based on four items and ranges from 0 to 6. These four items include Child‐Pugh classification (A = 0, B = 1, C = 2), tumor morphology (uninodular, ≤50%, 0; multinodular, ≤50%, 1; massive or >50%, 2), serum AFP level (<400 ng/mL, 0; ≥400 ng/mL, 1) and presence of portal vein thrombosis (no, 0; yes, 1).^2^

### 2.6 Definition of preoperative portal hypertension

When patients had detectable esophageal varices or splenomegaly and/or a platelet count below 10.0 × 10^4^/mm^3^, portal hypertension was diagnosed.^22^

### 2.7 Definition of TNM stage

In the present study, TNM Classification of Malignant Tumors, 8th edition, edited by the Union for International Cancer Control (UICC) was used for determining the TNM stage.^23^

### 2.8 Postoperative surveillance

Surveillance after surgery was carried out every 3 months. Serum AFP and PIVKA‐II level were routinely monitored every 3 months after surgery. Computed tomography (CT) was done every 3 months or when the levels of tumor markers were above the normal range. However, if 5 years had passed since surgery, CT interval was prolonged from 6 to 12 months, or CT was carried out when the levels of tumor markers were above the normal range.

### 2.9 Statistical analysis

All statistical analyses were carried out using SPSS software (version 23.0; IBM Co., New York, NY, USA). Significant statistical difference was defined as a *P* value of <0.05. Median and interquartile range were shown. Differences of the characteristics between the two LMR groups (<4.35 vs ≥4.35) were investigated with the chi‐squared test and the Mann‐Whitney *U* test. Hazard ratios (HR) with 95% confidence intervals (CI) were calculated by univariate or multivariate analyses using the Cox proportional hazard model. In order to determine the characteristics that were closely related to OS, multivariate analysis was carried out using the results of the univariate analyses. Kaplan‐Meier analysis and log‐rank test were used to compare OS and relapse‐free survival (RFS) of the two LMR groups (<4.35 vs ≥4.35).

## 3 RESULTS

Table 1 shows the CLIP score. One hundred and forty‐eight patients with high LMR value (≥4.35) and 181 patients with low LMR value (<4.35) were enrolled in the present study. Categorical clinical characteristics of the patients in the two LMR groups are listed in Table 2. There were no significant differences between the two LMR groups in all categorical clinical characteristics.

**Table 1:** The ROC curve of LMR for predicting OS in HCC patients with low CLIP score

Variable	Score		
	0	1	2
Child‐Pugh classification	A	B	C
Tumor morphology	Uninodular and extension ≤50%	Multinodular and extension ≤50%	Massive or extension >50%
AFP (ng/dL)	<400	≥400	
Portal vein thrombosis	No	Yes	

AFP, alpha‐fetoprotein; CLIP, Cancer of the Liver Italian Program ; HCC, hepatocellular carcinoma; LMR, lymphocyte‐to‐monocyte ratio; OS, overall survival; ROC, receiver operating characteristic.

**Table 2:** Relationships between categorical clinical characteristics and LMR in HCC patients with CLIP score 0 or 1

Variable	LMR ≥4.35 (n = 148) (45.0%)	LMR <4.35 (n = 181) (55.0%)	*P* value
Anatomical resection			
Absence	42 (12.8%)	59 (17.9%)	0.409
Presence	106 (22.2%)	122 (37.1%)	
Child‐Pugh classification			
A	130 (39.5%)	151 (45.8%)	0.259
B	18 (5.5%)	30 (9.2%)	
CLIP score			
0	98 (29.8%)	116 (35.3%)	0.687
1	50 (15.2%)	65 (19.7%)	
Gender			
Female	33 (10.0%)	32 (9.7%)	0.295
Male	115 (35.0%)	149 (45.3%)	
HBsAg			
Negative	131 (39.8%)	161 (48.9%)	0.788
Positive	17 (5.2%)	19 (5.8%)	
Not available	0 (0.0%)	1 (0.3%)	
HCVAb			
Negative	56 (17.0%)	60 (18.2%)	0.417
Positive	92 (28.0%)	120 (36.5%)	
Not available	0 (0.0%)	1 (0.3%)	
Hepatic vein invasion			
Absence	140 (42.6%)	169 (51.4%)	0.813
Presence	2 (0.6%)	3 (0.9%)	
Not available	6 (1.8%)	9 (2.7%)	
Liver cirrhosis			
Absence	65 (19.8%)	84 (25.5%)	0.095
Presence	80 (24.3%)	85 (25.8%)	
Not available	3 (0.9%)	12 (3.7%)	
No. of tumors			
1	112 (34.0%)	144 (43.7%)	0.399
≥2	36 (11.0%)	37 (11.3%)	
Pathological differentiation			
Well	42 (12.8%)	46 (14.0%)	0.731
Moderate or poor	103 (31.3%)	123 (37.4%)	
Not available	3 (0.9%)	12 (3.6%)	
Portal hypertension			
Absent	97 (29.5%)	99 (30.1%)	0.057
Present	50 (15.2%)	79 (24.0%)	
Not available	1 (0.3%)	3 (0.9%)	
Portal vein invasion			
Absent	105 (31.9%)	122 (37.1%)	0.568
Present	38 (11.6%)	51 (15.5%)	
Not available	5 (1.5%)	8 (2.4%)	
Surgery			
Laparoscopic	7 (2.1%)	3 (0.9%)	0.106
Open	141 (42.9%)	178 (54.1%)	
TNM stage			
I	85 (25.8%)	104 (31.6%)	0.944
II	56 (17.0%)	67 (20.4%)	
III	7 (2.2%)	10 (3.0%)	

Chi‐squared test was used for statistically analyzing the relationships between clinical characteristics and LMR.

CLIP, Cancer of The Liver Italian Program; HBsAg, hepatitis B surface antigen; HCC, hepatocellular carcinoma; HCVAb, hepatitis C virus antibody; LMR, lymphocyte‐to‐monocyte ratio; TNM, tumor‐node‐metastasis.

Continuous clinicolaboratory characteristics of patients in the two LMR group are listed in Table 3. There were significant differences between the two LMR groups in the serum levels of albumin, ALT and CRP.

**Table 3:** Relationships between continuous clinicolaboratory characteristics and LMR in HCC patients with CLIP score 0 or 1

Variable	LMR ≥4.35 (n = 148) (45.0%)	LMR <4.35 (n = 181) (55.0%)	*P* value
AFP (ng/mL)	14 (6‐53)	12 (5‐82)	0.732
Age (y)	67 (62‐74)	69 (62‐73)	0.447
Albumin (g/dL)	3.6 (3.3‐3.9)	3.5 (3.1‐3.9)	0.044
ALT (IU/L)	35 (25‐50)	29 (18‐44)	<0.001
AST (IU/L)	35 (27‐50)	35 (25‐46)	0.096
CRP (mg/dL)	0.10 (0.10‐0.21)	0.14 (0.10‐0.30)	0.006
ICGR15 (%)	14 (9‐19)	13 (9‐21)	0.935
Maximum tumor size (cm)	3.0 (2.0‐4.6)	3.0 (2.2‐5.0)	0.171
Observation period (d)	1507 (824‐1514)	1278 (626‐1902)	0.120
Platelet count (×10^4^/mm^3^)	14.0 (10.2‐17.5)	13.2 (9.5‐18.2)	0.534
PIVKA‐II (mAU/mL)	45 (24‐234)	65 (24‐437)	0.273
PT (%)	83 (76‐92)	82 (74‐90)	0.444
Total bilirubin (mg/dL)	0.6 (0.5‐0.8)	0.6 (0.5‐0.8)	0.674
WBC count (×10^3^/mm^3^)	5.0 (4.2‐6.0)	4.6 (3.8‐5.8)	0.068

Median (IQR), Mann‐Whitney *U* test.

AFP, alpha‐fetoprotein; ALT, alanine aminotransferase; AST, aspartate aminotransferase; CLIP, Cancer of the Liver Italian Program; CRP, C‐reactive protein; HCC, hepatocellular carcinoma; ICGR15, indocyanine green retention rate at 15 min; LMR, lymphocyte‐to‐monocyte ratio; PIVKA‐II, protein induced by vitamin K antagonist II; PT, prothrombin time; WBC, white blood cell.

Results of univariate analyses showed that AFP (>20/≤20, ng/mL), albumin (<3.5/≥3.5, g/dL), anatomical resection (presence/absence), Child‐Pugh classification (B/A), CRP (>0.3/≤0.3, mg/dL), ICGR15 (>13/≤13, %), LMR (<4.35/≥4.35), maximum tumor size (>3.5/≤3.5, cm), number of tumors (≥2/1), pathological differentiation (moderate or poor/well), platelet count (<14/≥14, ×10^4^/mm^3^), PIVKA‐II (>100/≤100, mAU/mL), portal hypertension (presence/absence), portal vein invasion (presence/absence), PT (<75/≥75, %) and TNM stage (III/I, II) were associated with OS of HCC patients with low CLIP score (0‐1) (Table 4). Multivariate analysis showed that LMR (<4.35/≥4.35) (HR, 2.022; 95% CI, 1.141‐3.583; *P *=* *0.016) and portal vein invasion (HR, 2.410; 95%CI, 1.258‐4.618; *P *=* *0.008) were closely associated with poor OS (Table 4).

**Table 4:** Univariate and multivariate analyses in relation to overall survival

Variable	Univariate			Multivariate		
	*P* value	HR	95% CI	*P* value	HR	95% CI
AFP (>20/≤20, ng/mL)	0.001	2.259	1.422‐3.590	0.230	1.425	0.799‐2.540
Age (>65/≤65, y)	0.633	1.121	0.702‐1.791			
Albumin (<3.5/≥3.5, g/dL)	<0.001	3.514	2.186‐5.650	0.070	1.778	0.954‐3.315
ALT (>50/≤50, IU/L)	0.313	1.333	0.763‐2.330			
Anatomical resection (Presence/Absence)	0.006	0.508	0.313‐0.824	0.434	0.767	0.396‐1.489
AST (>36/≤36, IU/L)	0.187	1.359	0.861‐2.143			
Child‐Pugh classification (B/A)	<0.001	3.909	2.066‐7.396	0.401	1.480	0.593‐3.695
CRP (>0.3/≤0.3, mg/dL)	0.015	1.953	1.139‐3.350	0.200	1.588	0.783‐3.220
Gender (Male/Female)	0.463	1.244	0.694‐2.231			
HBsAg (Presence/Absence)	0.881	0.946	0.454‐1.969			
HCVAb (Presence/Absence)	0.321	1.277	0.788‐2.070			
Hepatic vein invasion (Presence/Absence)	0.258	2.832	0.466‐17.20			
ICGR15 (>13/≤13, %)	<0.001	2.387	1.489‐3.826	0.054	1.868	0.990‐3.524
Liver cirrhosis (Presence/Absence)	0.593	1.136	0.712‐1.812			
LMR (<4.35/≥4.35)	0.031	1.667	1.047‐2.654	0.016	2.022	1.141‐3.583
Maximum tumor size (>3.5/≤3.5, cm)	0.001	2.222	1.396‐3.538	0.080	1.859	0.929‐3.720
Number of tumors (≥2/1)	0.007	2.064	1.214‐3.507	0.090	1.935	0.901‐4.155
Pathological differentiation (moderate or poor/well)	0.015	1.997	1.142‐3.491	0.072	1.885	0.945‐3.760
Platelet count (<14/≥14, ×10^4^/mm^3^)	0.004	1.880	1.227‐2.882	0.059	2.049	0.972‐4.318
PIVKA‐II (>100/≤100, mAU/mL)	0.001	2.483	1.340‐3.388	0.258	1.444	0.764‐2.729
Portal hypertension (Presence/Absence)	0.030	1.672	1.475‐4.043	0.171	0.612	0.303‐1.236
Portal vein invasion (Presence/Absence)	0.001	2.442	1.831‐4.489	0.008	2.410	1.258‐4.618
PT (<75/≥75, %)	0.001	2.000	1.197‐3.341	0.352	0.533	0.141‐2.009
TNM stage (III/I, II)	0.038	2.857	1.057‐7.721	0.854	1.073	0.508‐2.265
Total bilirubin (>0.7/≤0.7, mg/dL)	0.707	1.091	0.694‐1.713			
WBC count (>4.3/≤4.3, ×10^3^/mm^3^)	0.237	1.334	0.827‐2.153			

95% CI, 95% confidence interval; AFP, alpha‐fetoprotein; ALT, alanine aminotransferase; AST, aspartate aminotransferase; CLIP, Cancer of the Liver Italian Program; CRP, C‐reactive protein; HBsAg, hepatitis B surface antigen; HCVAb, hepatitis C virus antibody; HR, hazard ratio; ICGR15, indocyanine green retention rate at 15 min; LMR, lymphocyte‐to‐monocyte ratio; PIVKA‐II, protein induced by vitamin K antagonist II; PT, prothrombin time; TNM, tumor‐node‐metastasis; WBC, white blood cell.

Results of univariate analyses showed that AFP (>20/≤20, ng/mL), albumin (<3.5/≥3.5, g/dL), anatomical resection (presence/absence), Child‐Pugh classification (B/A), hepatitis C virus antibody (HCVAb) (Presence/Absence), ICGR15 (>13/≤13, %), LMR (<4.35/≥4.35), maximum tumor size (>3.5/≤3.5, cm), number of tumors (≥2/1), platelet count (<14/≥14, ×10^4^/mm^3^), PIVKA‐II (>100/≤100, mAU/mL), portal hypertension (presence/absence), PT (<75/≥75, %) and TNM stage (III/I, II) were associated with RFS of HCC patients with low CLIP score (0‐1) (Table 5). Multivariate analysis for RFS showed that LMR (<4.35/≥4.35) (HR, 1.804; 95% CI, 1.083‐3.005; *P *=* *0.024) was closely associated with poor RFS as well as HCVAb (presence/absence) (HR, 1.977; 95% CI, 1.144‐3.419; *P *=* *0.015) (Table 5).

**Table 5:** Univariate and multivariate analyses in relation to relapse‐free survival

Variable	Univariate			Multivariate		
	*P* value	HR	95% CI	*P* value	HR	95% CI
AFP (>20/≤20, ng/mL)	0.044	1.607	1.013‐2.548	0.253	1.383	0.794‐2.410
Age (>65/≤65, y)	0.379	1.228	0.777‐1.940			
Albumin (<3.5/≥3.5, g/dL)	0.001	2.302	1.431‐3.702	0.401	1.295	0.709‐2.367
ALT (>50/≤50, IU/L)	0.152	1.539	0.853‐2.778			
Anatomical resection (Presence/Absence)	0.019	0.544	0.327‐0.906	0.841	0.938	0.502‐1.752
AST (>36/≤36, IU/L)	0.153	1.390	0.885‐2.185			
Child‐Pugh classification (B/A)	0.001	4.143	1.796‐9.559	0.061	2.513	0.959‐6.587
CRP (>0.3/≤0.3, mg/dL)	0.270	1.374	0.781‐2.418			
Gender (Male/Female)	0.045	1.756	1.012‐3.049			
HBsAg (Presence/Absence)	0.107	0.565	0.282‐1.132			
HCVAb (Presence/Absence)	0.004	1.992	1.250‐3.173	0.015	1.977	1.144‐3.419
Hepatic vein invasion (Presence/Absence)	0.465	2.274	0.251‐20.59			
ICGR15 (>13/≤13, %)	0.003	2.015	1.275‐3.182	0.261	1.377	0.789‐2.404
Liver cirrhosis (Presence/Absence)	0.068	1.534	0.969‐2.429			
LMR (<4.35/≥4.35)	0.009	1.832	1.165‐2.878	0.024	1.804	1.083‐3.005
Maximum tumor size (>3.5/≤3.5, cm)	0.029	1.686	1.054‐2.695	0.071	1.755	0.952‐3.234
Number of tumors (≥2/1)	0.002	2.576	1.403‐4.729	0.080	1.946	0.923‐4.104
Pathological differentiation (moderate or poor/well)	0.162	1.432	0.866‐2.369			
Platelet count (<14/≥14, ×10^4^/mm^3^)	0.006	1.877	1.193‐2.952	0.190	1.522	0.812‐2.852
PIVKA‐II (>100/≤100, mAU/mL)	0.034	1.656	1.038‐2.643	0.230	1.438	0.794‐2.604
Portal hypertension (Presence/Absence)	0.001	2.207	1.361‐3.577	0.703	1.133	0.596‐2.152
Portal vein invasion (Presence/Absence)	0.076	1.621	0.950‐2.764			
PT (<75/≥75, %)	0.053	1.715	0.994‐2.961			
TNM stage (III/I, II)	0.040	4.776	1.073‐21.25	0.380	2.155	0.388‐11.97
Total bilirubin (>0.7/≤0.7, mg/dL)	0.424	1.221	0.749‐1.989			
WBC count (>4.3/≤4.3, ×10^3^/mm^3^)	0.636	1.118	0.704‐1.776			

95% CI, 95% confidence interval; AFP, alpha‐fetoprotein; ALT, alanine aminotransferase; AST, aspartate aminotransferase; CLIP, Cancer of the Liver Italian Program; CRP, C‐reactive protein; HBsAg, hepatitis B surface antigen; HCVAb, hepatitis C virus antibody; HR, hazard ratio; ICGR15, indocyanine green retention rate at 15 min; LMR, lymphocyte‐to‐monocyte ratio; PIVKA‐II, protein induced by vitamin K antagonist II; PT, prothrombin time; TNM, tumor‐node‐metastasis; WBC, white blood cell.

**Figure 2:**
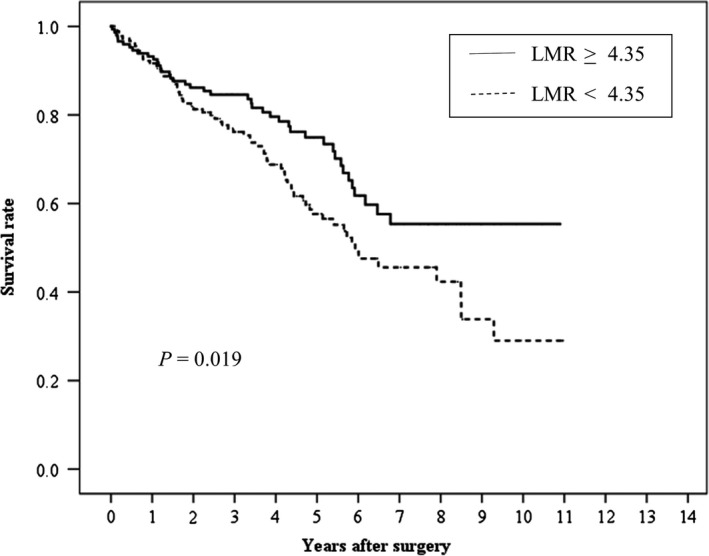
Relationship between the lymphocyte‐to‐monocyte ratio (LMR) and overall survival after curative resection for hepatocellular carcinoma patients with a Cancer of the Liver Italian Program (CLIP) score of 0 or 1

**Figure 3:**
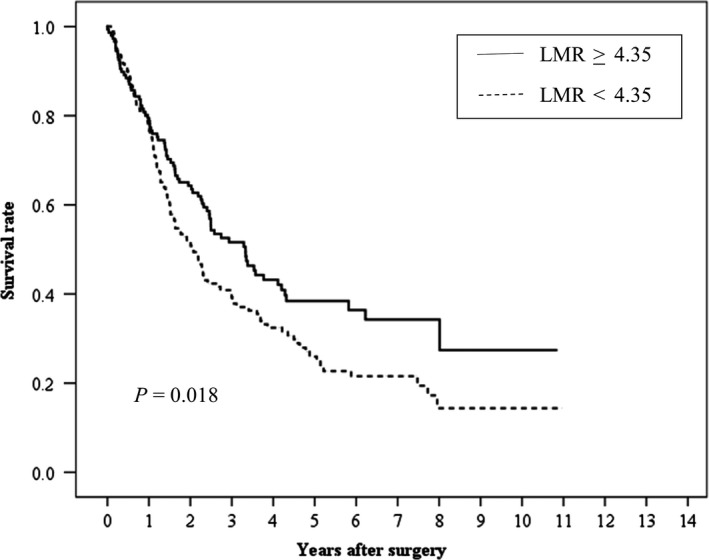
Relationship between the lymphocyte‐to‐monocyte ratio (LMR) and relapse‐free survival after curative resection for hepatocellular carcinoma patients with a Cancer of the Liver Italian Program (CLIP) score of 0 or 1

Median and maximum follow‐up periods for the survivors were 1379 days and 4004 days, respectively, with a mean survival period of 1454 ± 928 days. Kaplan‐Meier analysis and log‐rank test showed a significant difference in OS (Figure 2, *P* = 0.019) and RFS (Figure 3, *P* = 0.018) according to LMR (≥4.35 vs <4.35), respectively.

One hundred and sixteen patients died during the observation period. Among them, 76 patients died of HCC, 23 patients died of liver failure and the causes of death for the other 17 patients are as follows: two as a result of acute myocardial infarction, one as a result of heart failure, one as a result of hematemesis, two as a result of cerebral apoplexy, one as a result of cerebral infarction, two as a result of chronic renal failure, five as a result of pneumonia and three due to unknown causes.

**Figure 4:**
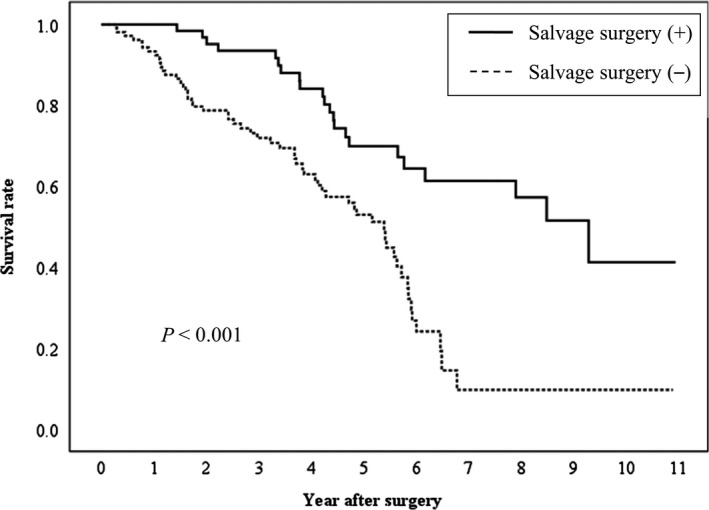
Relationship between salvage surgery and overall survival for hepatocellular carcinoma patients with a Cancer of the Liver Italian Program (CLIP) score of 0 or 1 who had intrahepatic recurrence after surgery

Among 329 patients who were enrolled in the present study, 169 patients (51.3%, 169/329) developed intrahepatic recurrence after liver resection. Among them, 63 patients (37.2%, 63/169) underwent salvage surgery. Kaplan‐Mayer analysis and log‐rank test showed that patients with salvage surgery for HCC recurrence had significantly better OS than those without salvage surgery (*P* < 0.001) (Figure 4). Results of chi‐squared test showed that LMR was not significantly associated with recurrence pattern (intrahepatic/extrahepatic) and salvage surgery (yes/no) (Table 6).

**Table 6:** Relationships between postoperative clinical characteristics and LMR in HCC patients with CLIP score 0 or 1

Variable	LMR ≥4.35 (n = 73) (38.8%)	LMR <4.35 (n = 115) (61.2%)	*P* value
Recurrence pattern			
Intrahepatic	66 (35.1%)	103 (54.8%)	0.851
Extrahepatic	7 (3.7%)	12 (6.4%)	
Salvage surgery			
Yes	49 (26.1%)	76 (40.5%)	0.883
No	24 (12.7%)	39 (20.7%)	

Chi‐squared test was used for statistically analyzing the relationships between postoperative clinical characteristics and LMR.

CLIP, Cancer of the Liver Italian Program; HCC, hepatocellular carcinoma; LMR, lymphocyte‐to‐monocyte ratio.

## 4 DISCUSSION

It has been reported previously that most patients who undergo curative liver resection for HCC have a low CLIP score (0‐1) (73.4%, 433/599)^6^. In order to apply the CLIP score to prognostication after curative surgery for HCC, a score of 0 or 1 must stratify postoperative outcome. However, because the CLIP score is unable to stratify the postoperative outcome of HCC patients with a low score (0‐1),^5^ its performance for prognostication after curative surgery for HCC is poor. Our study showed that the LMR (<4.35/≥4.35) significantly stratified the outcome after curative surgery for HCC patients with a low CLIP score, thus resolving this problem associated with the CLIP score.

Although previous studies have shown that a low LMR is associated with tumor progression,^16^, ^17^, ^18^ we found that a low LMR (<4.35) was not associated with tumor‐related characteristics such as vascular invasion, TNM stage, tumor markers and tumor size. However, both OS and RFS in the low‐LMR group were significantly poorer than those in the high‐LMR group, as has been shown previously.^16^, ^17^, ^18^ Because cancer is the main cause of death in HCC patients, and RFS in the low‐LMR group was poorer than that in the high‐LMR group, the poor OS in the low‐LMR group might have been as a result of recurrence of HCC, which was more frequent than in the high‐LMR group.

However, because HCC recurrence can be divided into two types—intrahepatic metastasis (IM) and multicentric occurrence (MO),^24^—it is unclear whether HCC recurrence is one type or the other. IM is defined as intrahepatic recurrence from the original HCC. However, MO occurrence is defined as new intrahepatic occurrence of HCC. A previous study has reported that positivity for HCVAb, a preoperative low platelet count and large tumor size were useful for the diagnosis of HCC recurrence as a result of IM.^25^ Because LMR was not associated with HCVAb, platelet count or tumor size, our result indicated that LMR might be related to MO. In addition, early recurrence of HCC arises mainly from IM and late recurrence is more likely to be caused by MO in origin.^26^ Previous study has reported that a recurrence‐free time of 3 years after surgery was a significant cut‐off time for distinguishing between IM and MO.^27^ RFS curve in the present study indicated that early recurrence of patients with low LMR (<4.35) was higher than that with high LMR (≥4.35). This fact indicated that the proportion of patients with IM recurrence might be strongly associated with a low‐LMR group as compared to a high‐LMR group, because the RFS rate of a low‐LMR group decreased more rapidly in the early phase than that of a high‐LMR group. In view of the retrospective nature of our study, however, it was unclear whether HCC recurrence had been IM or MO recurrence. Further study will be necessary to determine whether low LMR is associated with HCC recurrence as a result of MO recurrence.

Hepatitis viral infection and liver cirrhosis are both important risk factors for development of HCC.^28^, ^29^ In fact, most of our patients had HCV infection (64.5%, 212/329) and liver cirrhosis (50.1%, 165/329). Although the relationship between LMR and carcinogenesis of HCC is unknown, two studies have suggested that a low LMR might reflect suppression of host immunity as a result of the tumor.^16^, ^18^ MO recurrence was frequently observed in patients with chronic hepatitis among the low‐LMR group. Therefore, although LMR was not associated with tumor‐related characteristics, a low LMR was closely associated with poor OS and RFS in the present study.

Although previous studies did not investigate the serum levels of albumin and CRP as prognostic factors,^16^, ^17^, ^18^ our present study showed that LMR was significantly associated with the serum levels of albumin and CRP. Albumin and CRP commonly indicate the nutritional status of cancer patients.^30^ In fact, in cancer patients, malnutrition is significantly correlated with a low lymphocyte count, and this can have an adverse impact on prognosis. Because our study showed that patients with a low LMR had poorer nutritional status than those with a high LMR, nutritional intervention might improve the prognosis of such patients.^30^, ^31^ In fact, a previous study has shown that nutritional therapy can improve the outcome after surgery for HCC.^32^ Therefore, further studies of the relationship between LMR and nutrition in HCC patients are warranted.

The LMR can significantly stratify postoperative outcome in HCC patients with a low CLIP score undergoing curative resection, and combination of the LMR with the CLIP score was able to predict the postoperative outcome after curative resection for HCC. Although postoperative intervention is needed for patients with a low LMR to prevent HCC recurrence, there is no effective postoperative adjuvant chemotherapy for such patients. A recent study showed that sorafenib is not an effective intervention in the adjuvant setting for HCC patients following resection or ablation.^33^ Among other postoperative adjuvant therapies for HCC patients, immunotherapy, interferon therapy and internal radiation therapy have been reported.^34^, ^35^, ^36^ A meta‐analysis has concluded that interferon therapy is effective for prevention of HCC recurrence after surgery.^37^ However, patients rarely receive interferon therapy because it has various restrictions such as age (≤70 years), performance status (≤2), Child‐Pugh score (≤7), platelet count (≥10.0 × 10^4^/mm^3^) and WBC count (≥3.0 × 10^3^/mm^3^). Additionally, interferon therapy is very costly. Because there is no appropriate indication for adjuvant therapy after surgery for HCC patients, early detection of recurrence through tight postoperative surveillance is necessary for HCC patients with a low CLIP score (0‐1) and a low LMR (<4.35), in order to improve survival after surgery.

In conclusion, we have carried out a retrospective database analysis at a single institution to investigate the relationship between the LMR and outcome in HCC patients with a low CLIP score undergoing curative resection. Our results showed that LMR significantly stratified the postoperative survival of those patients who had a low CLIP score.

## DISCLOSURE

Ethical Statement: This study complied with the standards of the Declaration of Helsinki and the current ethical guidelines and was approved by the institutional ethical board of our institution (provided ID number: 28068).

Conflicts of Interest: Authors declare no conflicts of interest for this article.
